# Diet Quality and Its Relationship with Antioxidant Status in Patients with Rheumatoid Arthritis

**DOI:** 10.1155/2018/8506343

**Published:** 2018-04-05

**Authors:** Anna Prescha, Katarzyna Zabłocka-Słowińska, Sylwia Płaczkowska, Daiva Gorczyca, Anna Łuczak, Marcelina Majewska, Halina Grajeta

**Affiliations:** ^1^Department of Food Science and Dietetics, Wrocław Medical University, Borowska 211, 50-556 Wrocław, Poland; ^2^Diagnostics Laboratory for Teaching and Research, Wrocław Medical University, Borowska 211, 50-556 Wrocław, Poland; ^3^3rd Department and Clinic of Paediatrics, Immunology and Rheumatology of Developmental Age, Wrocław Medical University, Koszarowa 5, 51-149 Wrocław, Poland; ^4^Department and Clinic of Rheumatology and Internal Medicine, Wrocław Medical University, Borowska 213, 50-556 Wrocław, Poland; ^5^Student Scientific Club Food Science and Dietetics, Wrocław Medical University, Wrocław, Poland

## Abstract

A direct contribution towards destructive, proliferative synovitis in rheumatoid arthritis (RA) has been attributed to reactive oxygen species action. Some nutrients are considered to be capable of improving the oxidant/antioxidant status in RA; however the impact of diet composition on the antioxidant capacity of serum has not yet been studied in this disease. The aim of the study was to assess the relationship between diet quality and antioxidant status in patients with RA and healthy controls. Nutritional assessment was performed, and antioxidant status in serum, without and with deproteinization (TAS and DSAS, resp.), was determined in 82 RA and 87 healthy subjects. The diet of the RA group was low-energy and imbalanced. TAS and DSAS were significantly lower in RA patients than in controls. Antioxidant status significantly correlated with the supply of foods and nutrients influencing antioxidant and anti-inflammatory defense in RA; however, in this group, TAS was more sensitive to diet than DSAS. In healthy subjects, the nonprotein pool of serum antioxidants was more tightly linked to diet. These outcomes indicate the need to monitor diet quality of patients with RA and the usefulness of TAS measurements in this monitoring.

## 1. Introduction

Rheumatoid arthritis (RA) is a chronic inflammatory autoimmune disease, causing destruction of the joints, which consequently leads to disability and may also affect the life span [1]. A direct contribution towards destructive, proliferative synovitis in RA has been attributed to reactive oxygen species action [2, 3]. Markers of oxidative stress in the organism, such as blood and urine lipid peroxidation products, levels of reactive oxygen and nitrogen species, and total oxidant status, all increase in patients suffering from this disease [4–7]. Oxidative stress affects extracellular antioxidant concentrations, as well as the antioxidant enzyme activities measured in whole blood and serum of RA patients [8–11]. The total serum antioxidant status in RA, measured using different methods, has been found to be lower than those in healthy subjects. The levels of antioxidants in the blood and total antioxidant status negatively correlate with disease activity and duration; moreover, a low concentration of antioxidant components in the blood has been recognized as a risk factor for RA [4, 12, 13].

Several studies have shown that an improvement in total antioxidant/oxidant status and mitigation of disease activity could be attained by antioxidant vitamins, polyphenols, and selenium supplementation at relatively high doses, although this high intake was not reflected in the concentration of those antioxidants in the blood of the patients [5, 14, 15]. The intake of selected dietary antioxidants (tocopherols, carotenoids, vitamin C, and selenium) was not found to correlate with their serum level in RA patients either, and furthermore, oxidative stress as measured by MDA level was not affected by the intake of these compounds [16]. As diet supplies various compounds of antioxidant activity (including polyphenols) and those acting synergistically to antioxidants, measurements of selected dietary compounds may not reflect the total impact of diet on antioxidant/oxidant status. Moreover, in response to oxidative stress, adaptive mechanisms in the organism are initiated, which are considered to contribute to the misrepresentation of dietary antioxidant effects in the body [6]. The relationship between serum total antioxidant status (TAS) and the quality and composition of the diet has not yet been studied in RA patients. Recently, it has been demonstrated that diet quality may contribute to the course and activity of RA [17]. Evaluation of diet quality and its relationship with antioxidant status in patients with RA may help indicate dietary habits and nutrient intakes relevant to the improvement of antioxidant defense, and thereby to the mitigation of symptoms associated with RA [14].

A number of methods are used to measure serum antioxidant capacity as a marker of the condition of the antiradical defense system. Among them, a method with 2,2′-azino-bis(3-ethylbenzothiazoline-6-sulfonic acid) diammonium salt (ABTS reagent) has been established (Randox method). The ABTS test measures the ability of nonenzymatic serum components to reduce the radical cation of ABTS [18, 19]. In human plasma, TAS is mainly conditioned by albumin (representing 43–53% of the total value) and uric acid (33%). It has been shown that TAS additionally includes ascorbic acid, *α*-tocopherol, and bilirubin activity [20]. The rheumatoid cachexia occurring in RA patients may affect protein antioxidant level in the serum and thereby distort assessment of the impact of dietary components on antioxidant status [21]. Therefore, in addition to TAS determination, serum antioxidant status as measured after deproteinization in organic solutions (acetonitrile and methanol) using 2,2′-diphenyl-1-picrylhydrazyl (DPPH) stable radical may be proposed as potentially useful in the assessment of diet relation to antioxidant status in RA. DSAS method has been shown to be sensitive to the negligible differences in serum level of ascorbic acid, quercetin, and uric acid in healthy subjects [22]. We hypothesize that measurements of deproteinized serum antioxidant status (DSAS) may highlight the antiradical properties of hydrophilic and lipophilic nutrients; hence, we can better visualize the relationship of diet with the antioxidant status of RA patients.

The aim of this study was to assess diet quality and estimate the impact of diet composition on the antioxidant status, as measured in serum without and with deproteinization (TAS and DSAS, resp.), of patients with RA and healthy subjects.

## 2. Patients and Methods

Eighty-two patients with RA and 87 healthy controls were included in the study. Participants were recruited via the outpatient clinic of the Department of Rheumatology and Internal Medicine, Wrocław Medical University, and all patients with RA fulfilled the American College of Rheumatology criteria. The control group consisted of healthy people recruited from public offices and Wrocław 3rd Age Universities. Exclusion criteria for the control group were proinflammatory diseases and mental health issues. The study protocol was approved by the Wrocław Medical University Ethics Board (consent no. KB-390/2012). All patients gave written informed consent.

The patients with RA underwent clinical examination by a rheumatologist, and the following data were collected: age when consent for the study was given, duration of disease, number of swollen joints, number of painful joints, the presence of rheumatoid factor (RF), anticitrullinated protein antibodies (ACPA), the Disease Activity Score of 28 joints (DAS 28), and pharmacological treatment. In both RA and control groups, body mass index (BMI) was measured, and data on declared smoking status were collected.

For diet quality assessment, the study used 3-day food records and a dietary habits questionnaire developed at the Department of Food Science and Dietetics, Wrocław Medical University [23]. The *Album of Photographs of Food Products and Dishes* was used to estimate food portion sizes [24]. The questionnaire consisted of 106 questions concerning the consumption of food groups, as well as individual foods within the groups calculated in portions per unit of time. The calculation of the composition of daily food rations was made using the computer program Diet 5.0 developed by the National Food and Nutrition Institute in Warsaw [25].

Blood samples were collected from all subjects under fasting conditions, and TAS was determined by the spectrophotometric method with 2,2′-azino-bis(3-ethylbenzothiazoline-6-sulfonic acid) diammonium salt (ABTS reagent, Randox Total Antioxidant Status, Randox Laboratories Ltd., cat. no. NX 2332) using autoanalyzer Konelab 20i (Thermo Fisher Scientific, USA) [20]. DSAS was also measured spectrophotometrically with DPPH radical using Spectronic GENESYS 6 UV-visible spectrophotometer (Thermo Electron Corporation, USA) [22]. In both methods, antioxidant status was expressed in Trolox equivalents. Serum albumin level was determined as a coloured complex with bromocresol green, and uric acid—based on oxidation with uricase. These measurements were performed with the autoanalyzer Konelab 20i.

Statistical calculations were performed using Statistica StatSoft 12.0. Pearson's *χ*^2^ test was used for assessment of differences in qualitative variables between groups. The correlation of antioxidant status with nutrient intake and frequency of food consumption was assessed in both RA and control groups, further divided into current smoker and nonsmoker (former and never smoker) subgroups. Depending on the distribution of variables, either Student's *t*-test or the Mann–Whitney *U* test was used for group comparisons, and Pearson or Spearman correlation analysis was performed for measurements of association between variables.

## 3. Results

The baseline characteristics of the patients and control subjects are summarised in Table 1. The RA group consisted of a higher percentage of female patients and a lower percentage of current smokers in comparison with the control group (*p* < 0.02). More than half of the RA and control subjects were overweight or obese. Significantly lower (*p* < 0.000001) concentration of serum albumin was found in RA patients in comparison to control subjects; however, the median of albumin value in the RA group did not indicate protein malnutrition (<3.5 g/dL). For the majority of patients, RA therapy included steroids and also methotrexate.

**Figure 1:**
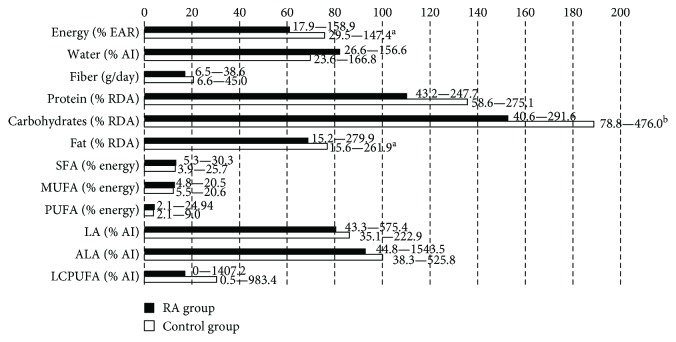
Macronutrient intake and dietary fat components in RA and control groups (% of recommendations)—median and range. SFA: saturated fatty acids; MUFA: monounsaturated fatty acids; PUFA: polyunsaturated fatty acids: LA: linoleic acid; ALA: *α*-linolenic acid; LCPUFA: long chain polyunsaturated fatty acids; EAR: estimated average requirement; AI: adequate intake; RDA: recommended dietary allowances. Statistically significant differences (*p* value): ^a^<0.005, ^b^<0.0001.

**Figure 2:**
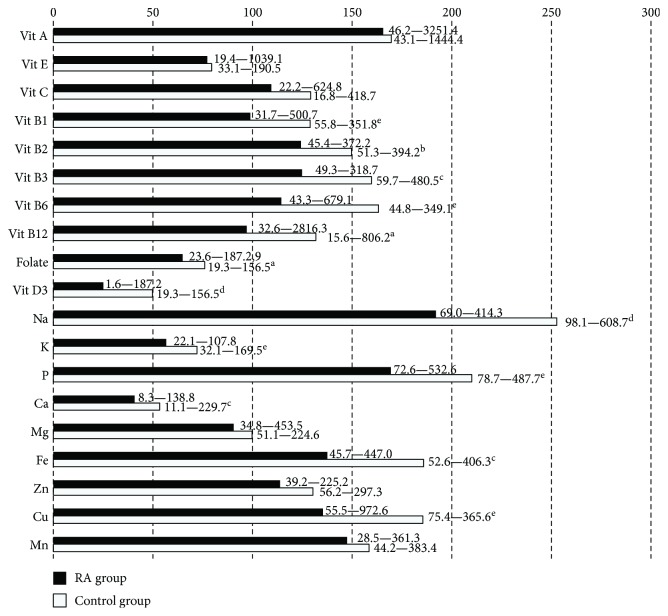
Micronutrient intake in RA and control groups (% of recommendations)—median and range. The values expressed in % RDA, except for vitamin E, Na, and K (% AI) and Mn (mean AI in European countries). Statistically significant differences (*p* value): ^a^<0.05, ^b^<0.01, ^c^<0.005, ^d^<0.001, ^e^<0.0001.

The assessment of dietary intake using food records showed that the diet of subjects in both groups was low-energy and provided an insufficient amount of fat; moreover, a significantly lower intake of these nutrients was observed in the RA than in the control group (*p* < 0.002, Figure 1). In both groups, an insufficient intake of long chain polyunsaturated fatty acids (LCPUFA) and vitamin E was shown. The amount of dietary folate and vitamin D was also low, especially in the RA group (*p* = 0.02 and *p* = 0.00008, resp.). An excessive supply of sodium, phosphorus, iron, copper, and manganese and a very low intake of potassium and calcium were observed in both groups (Figure 2).

Diet quality assessment also included an analysis of food consumption frequencies in both groups. As the comparison of dietary habits between RA and the control group generated numerous data, only the consumption frequencies of food groups rich in antioxidants were presented in Table 2. It has been shown that levels of consumption of vegetables, fruits, and fruit and vegetable juices did not differ significantly between groups and in the majority of subjects did not exceed 2 servings per day (Table 2). Chocolate, dried fruits, and tea were consumed significantly more often in the RA group (*p* < 0.02), whereas whole-grain cereal products, coffee, herbs, and spices were eaten more rarely (*p* < 0.02). Around half of the subjects in both groups consumed vegetable oils irregularly.

Measurements of serum antioxidant status showed significantly lower values of TAS, as measured using ABTS reagent, in patients with RA when compared to the control group (median 1.47 versus 1.72 mM Trolox; *p* = 0.031, Table 3). DSAS, measured using the DPPH method, was also significantly lower in RA patients (median 174.3 versus 206.4, *p* = 0.027). A significant correlation between TAS and DSAS values was observed in both groups; however, this relationship was stronger in RA patients (*r* = 0.56, *p* = 0.019). TAS correlated positively with serum albumin concentration in the control group (*r* = 0.34, *p* < 0.000001), while in the RA group, a significant correlation (*r* = 0.23, *p* = 0.027) was found only in subjects with an albumin level equal to, or over, the median value (3.93 g/dL). In contrast, DSAS correlated negatively (*r* = −0.21, *p* = 0.049) with albumin level only in subjects with an albumin level lower than 3.93 g/dL. Both parameters used for antioxidant status assessment positively correlated with serum uric acid concentration, whether in RA (TAS: *r* = 0.58, *p* = 0.027; DSAS: *r* = 0.39, *p* = 0.017) or in healthy subjects (TAS: *r* = 0.62, *p* = 0.0011; DSAS: *r* = 0.27, *p* = 0.031). Moreover, TAS level was negatively related to the number of swollen joints (*r* = −0.37) and disease duration (*r* = −0.25) in RA patients (*p* < 0.03).

The associations between diet composition and serum antioxidant status were significant in both studied groups. TAS values positively correlated, especially in the RA group (*r* = 0.21 − 0.87, *p* < 0.04), with the supply of foods and nutrients considered important in antioxidant status maintenance (Table 4). A significant impact on TAS seemed to be attributed to the composition of dietary fat in RA patients. Smoking status considerably influenced the correlation of TAS with vegetables, groats, chocolate, and Ca supply in the diet. Moreover, in the case of meat and fish consumption, we observed strong yet opposing effects on TAS in nonsmoking RA patients. In the control group, only the intake of total fat, monounsaturated fatty acids (MUFA), and vitamin A, as well as the n-6/n-3 fatty acid ratio, positively influenced TAS values. DSAS values positively correlated with the consumption of whole-grain bread and legumes in both groups. In the RA group, a positive correlation was also discovered for frequency of tea consumption, while in the control group, for frequency of apple consumption. A significant relationship between DSAS and intake of unsaturated fatty acid, dietary fiber, and vitamin A, as well as Fe and Cu, was found in the control group (*r* = 0.21 − 0.55, *p* < 0.05), while in the case of vitamin C, the correlation concerned the nonsmoker subgroup only.

## 4. Discussion

The assessment of diet quality based on nutrient intake indicates that the diet of the RA group was low-energy, and not well balanced. The insufficient intake of energy was not reflected in BMI values of the studied RA population since the majority of them were overweight or obese. This confirms previous findings that BMI is not a reliable parameter to detect malnutrition in RA, due to the abnormality of fat/fat-free mass ratio as a result of RA cachexia [26, 27]. The impact of a low-energy diet on oxidant/antioxidant status has not yet been studied in RA or other autoimmune disorders. In overweight and normal-weight healthy individuals, as well as in subjects with metabolic syndrome, energy restriction is considered to contribute to oxidative stress reduction—however, this dietary modification needs to be accompanied by an adequate supply of essential nutrients [28, 29]. In the RA patients recruited to this study, the low supply of energy was mainly due to insufficient fat intake, especially n-3 PUFA (*α*-linolenic acid and LCPUFA). The deficiency of these fatty acids is unfavourable in RA, because of the proven beneficial effects of n-3 PUFA in the improvement of disease activity, inflammatory, and oxidative markers [30, 31]. Sodium intake, which has been shown to promote Th17 cell-mediated inflammation, particularly in smoking subjects [32], was almost twice the RDA value in the studied RA group. The assessment of the dietary habits of RA patients revealed that the consumption of foods involved in antioxidant status maintenance, especially whole-grain cereals, fruits, and vegetables, was low. Generally, patients with RA were more likely to have poorer diet quality than do healthy subjects. Inadequate diet quality, assessed using Healthy Eating Index (HEI) 2010, was also observed in RA patients by Berube et al. [17] with a prevalence of low whole-grain and dairy food consumption.

An impairment of antioxidant defense in RA patients was also shown in our study. The TAS values were lower in the RA than in the healthy population, confirming the findings of other authors [13, 33]. Likewise, the comparison of DSAS values between groups enabled us to detect lower antioxidant activity by nonprotein antiradicals in the RA group, when compared to the controls. In both RA and control groups, DSAS values comprised about 12% of Randox-TAS values, expressed as mM of Trolox equivalents in serum. According to the published data, the contribution of nonprotein antioxidants to serum TAS is higher and varies from 47 to 93% depending on the method used [20, 34]. Both DPPH and ABTS tests measure the capacity of antioxidant compounds for scavenging stable free radicals; however, the substrates and environments of reaction differ between methods. Organic solvents used in the DPPH test (methanol and acetonitrile) might negatively affect antioxidant activity in comparison with solutions containing water [35].

Impaired antioxidant status in the RA group may be related to the loss of antioxidant components due to the neutralisation of radicals produced as a consequence of inflammation in the joints involved, and this may be also caused by an inadequate supply of antioxidant nutrients [36, 37]. Depletion of the antioxidant pool in RA may be also a consequence of therapy [38]. In the studied RA group, an inverse correlation of TAS with disease duration and number of swollen joints was shown. In published studies, a positive correlation between biomarkers of oxidative stress and RA disease severity was shown; hence, these findings suggest that TAS value might serve as a valuable marker in the assessment of redox status and RA progression [39, 40]. The strong influence of endogenous antioxidant components, that is, albumin and uric acid, on the antioxidant status of serum without deproteinization observed in the control group was expressed to a lesser extent in the RA group. A strong relationship between TAS values and the concentration of uric acid levels in healthy subjects, as well as populations with different chronic diseases, has been previously shown in several studies [20, 41]. More than half of the RA group were treated with methotrexate, which could affect the level of uric acid and hence deplete the pool of low-molecular-weight antioxidants in serum [38]. The uric acid determination performed in our study, however, did not reveal significant differences between groups (*p* = 0.062). As in the RA group, a significantly lower concentration of serum albumin was found than that in the controls, indicating enhanced protein catabolism related to inflammatory processes [21]; hence, the contribution of this macromolecule to the serum antioxidant pool seems to be weaker than that of other components. The considerable impact of the nonprotein constituents of the antioxidant pool is confirmed by a not particularly strong, yet significant, negative correlation between albumin and DSAS values in RA patients with low albumins, suggesting the compensative role of micronutrients in the antioxidant defense of patients suffering from protein disturbances.

The analysis of the relationships between TAS and diet composition carried out in this study suggests that fat components, especially total PUFA, SFA, and vitamin E, play an important role in the antioxidant defense of RA patients. Among antioxidant vitamins, tocopherols were found to be effective in the inhibition of joint destruction in the animal model [42]; however, in patients with RA, an improvement in oxidation status and total disease severity stage was only observed in combination with ascorbic acid and vitamin A [15]. Pyridoxine was the only water-soluble vitamin to enhance TAS in our study, which may confirm the benefits of supplementing this vitamin as a part of RA treatment [43]. The strong effects of meat and fish consumption on TAS in the RA group may be attributed to the fat composition of these foods and its impact on antioxidant and anti-inflammatory status [44, 45]. In addition to this finding, the significant effects on TAS noticed in the RA group related to the consumption of foods rich in antioxidants, for example, vegetables, especially leafy ones, chocolate, and groats, lead us to emphasize the important role of diet in the maintenance of the antioxidant defense of patients suffering from this autoimmune disease. The correlation of fruit juices consumption with TAS noticed in this population could result from the fructose rather than the antioxidant content in these foods, as it has been shown that fructose utilisation significantly influences the level of uric acid comprising an important part of the antioxidant pool in serum [46, 47].

Determination of DSAS was used in this work to assess the total activity of exogenous low-molecular-weight antioxidants, such as vitamins and polyphenols, as well as endogenous uric acid. This study revealed considerably less correlations of DSAS with nutrient intake and food consumption in the RA group than in controls. A positive correlation of the DSAS test with many dietary nutrients, including fatty acids, vitamins, and minerals, was observed in the control group. The effect of PUFA on TAS was not confirmed for DSAS in the RA group. This may result from the involvement of fatty acid metabolites in the modulation of macromolecular components of inflammation, such as cytokines, rather than the microcomponents of the serum antioxidant pool [48]. Moreover, a negative effect of vitamin C intake on DSAS was observed, albeit ascorbic acid has recently been shown to significantly reduce reactive oxygen and nitrogen species levels in individuals with RA [5]. The positive correlation between tea consumption and DSAS values may confirm the beneficial role of tea polyphenols in RA [49].

It should be noted that the relationship between antioxidant status and certain dietary components was influenced by smoking status, especially in the RA group, indicating the strong impact of this environmental factor on oxidant/antioxidant ratio in RA. Significant depletion of antioxidant capacity has been previously shown in healthy smokers when compared to nonsmokers (a well-known factor) [50]. Moreover, in smokers suffering from RA, an increase of disease activity was observed [51].

## 5. Conclusions

The results of this study indicate the relationship between the serum antioxidant status of RA patients and the quality and composition of their diet. The disturbance of protein metabolism in RA seems to sensitize the antioxidant status of an affected organism to a diet which is imbalanced and poor in dietary antioxidants and anti-inflammatory agents.

We have shown the greater usefulness of TAS rather than DSAS measurements in the assessment of dietary impact on antioxidant capacity in RA patients; however, for healthy subjects, the nonprotein contribution in serum antioxidant capacity seems to be more sensitive to diet.

This study confirms the need to monitor the intake of nutrients involved in the antioxidant defense of patients with RA.

## Figures and Tables

**Table 1:** Group characteristics.

Parameter	RA group (*n* = 82)	Control group (*n* = 87)	*p* value
Sex of subjects, *n* (% of group)			
Female	61 (74.4%)	50 (57.5%)	0.0012
Male	21 (25.6%)	37 (42.5%)	
Age (years), median (range)	54 (20–82)	53 (25–78)	NS
BMI (kg/m^2^), *n* (%)			
<18.5	2 (2.4%)	2 (2.3%)	NS
18.5–24.9	36 (43.9%)	29 (33.3%)	
≥25	44 (53.7%)	56 (64.4%)	
Cigarette smoking, *n* (%)			
Current smoker	22 (26.8%)	31 (35.6%)	0.023
Former smoker (cessation ≥ 1 year)	29 (35.4%)	38 (43.7%)	
Never smoker	31 (37.8%)	18 (20.7%)	
Albumin (g/dL), median (range)	3.93 (2.57–5.70)	4.23 (2.85–5.42)	<0.000001
Uric acid (g/dL), median (range)	4.3 (1.5–9.1)	5.2 (3.0–8.8)	NS
Time from RA onset (years), median (range)	8.5 (0.2–50)	—	—
RF + (cutoff 20 IU/mL), *n* (%)	53 (64.6%)	—	—
ACPA + (cutoff 25 U/mL), *n* (%)	41 (50.0%)	—	—
Number of swollen joints, median (range)	4 (0–24)	—	—
DAS 28, median (range)	5.01 (1.49–8.49)	—	—
Treatment			—
Methotrexate	46 (56.1%)	—	—
Other nonbiologic DMARDs	33 (40.2%)	—	—
Anti-TNF therapy	23 (28.0%)	—	—
Steroids	62 (75.6%)	—	—

*n*: number of subjects; NS: not significant; Anti-TNF, anti-tumour necrosis factor; BMI: body mass index; RF: rheumatoid factor; ACPA: anti-citrullinated protein antibody; DAS 28: Disease Activity Score of 28 joints; DMARDs: disease-modifying antirheumatic drugs.

**Table 2:** Consumption of food sources of antioxidants.

Food consumption		RA (% of group)	Control (% of group)	*p* value
Whole-grain cereal products	≥3 servings/day	2.4	14.9	0.018
	1-2 servings/day	37.8	36.8	
	Irregularly	59.8	48.3	

Vegetables	≥3 servings/day	34.1	29.9	NS
	1-2 servings/day	58.5	63.2	
	Irregularly	7.3	6.9	

Fruits	≥3 servings/day	23.2	26.4	NS
	1-2 servings/day	65.9	62.1	
	Irregularly	11.0	11.5	

Fruit and vegetable juices	≥3 cups/day	4.9	1.1	NS
	1-2 cups/day	37.8	28.7	
	Irregularly	57.3	70.1	

Nuts, seeds, and legumes	>5 servings/week	12.2	4.6	0.019
	2–5 servings/week	14.6	28.7	
	≤1 serving/week	73.1	65.5	

Dried fruits	>5 servings/week	9.8	1.1	0.0011
	2–5 servings/week	22.2	6.9	
	≤1 serving/week	70.7	92.0	

Chocolate	≥15 g/day	14.6	6.9	0.0083
	4–14 g/day	42.7	34.5	
	Irregularly	48.7	58.6	

Tea	≥4 cups/day	25.6	12.6	0.000060
	2-3 cups/day	57.3	39.1	
	≤1 cups/day	17.1	48.3	

Coffee	≥4 cups/day	1.2	9.2	0.000052
	2-3 cups/day	13.4	47.1	
	≤1 cups/day	85.4	43.7	

Vegetable oils	>1 tablespoon/day	7.3	12.6	NS
	0.5–1 tablespoon/day	35.3	39.1	
	Irregularly	57.3	48.3	

Herbs and spices	≥0.5 teaspoon/day	6.1	11.5	0.0043
	<0.5 teaspoon/day	74.3	85.1	
	Irregularly	19.5	3.4	

NS: not significant.

**Table 3:** Serum antioxidant capacity (TAS and DSAS) level and significant correlations with clinical parameters.

	RA group	Control group	*p* value
Serum antioxidant capacity (mM Trolox)			
TAS, median (range)	1.47 (1.03–1.86)	1.72 (1.33–2.14)	0.031
DSAS, median (range)	174.3 (61.0–352.3)	206.4 (97.1–523.1)	0.027
TAS/DSAS correlation (*r* values)	0.56	—	0.019
	—	0.25	0.038
Significant correlation of TAS with clinical parameters (*r* values)			
Albumin	0.23^∗^	—	0.027
	—	0.52	<0.000001
Uric acid	0.58	—	0.027
	—	0.62	0.0011
Number of swollen joints	−0.37	—	0.022
Time from RA onset	−0.25	—	0.027
Significant correlation of DSAS with clinical parameters (*r* values)			
Albumin	−0.21^∗∗^	—	0.049
	—	0.31	0.0042
Uric acid	0.39	—	0.017
	—	0.27	0.031

^∗^Only in patients with albumin level ≥ 3.93 (median value in RA group). ^∗∗^Only in patients with albumin level < 3.93 (median value in RA group).

**Table 4:** TAS and DSAS correlation with food consumption and nutrient intake.

	RA group	Control group	*p* value
Significant correlation of TAS with food consumption/nutrient intake (*r* values)			
Meat	−0.76^∗^		0.0012
Fish	0.87^∗^		0.0013
Groats	0.73^∗^		0.0016
Vegetables (total)	0.42^∗∗^		0.011
Leafy vegetables	0.85^∗^		0.039
Cruciferous vegetables	0.41^∗∗^	—	0.018
	—	−0.39^∗∗^	0.0082
Fruit and vegetable juices	0.42		0.0073
Chocolate	0.43^∗∗^		0.0029
% energy from fat		0.27	0.033
% energy from LA	0.22		0.027
% energy from ALA	0.39		0.022
% energy from PUFA	0.28		0.037
% energy from MUFA		0.23	0.042
% energy from SFA	−0.38^∗∗^		0.0018
n-6/n-3 fatty acid ratio		0.20	0.046
% RDA of vitamin E	0.21		0.035
% RDA of vitamin A		0.22	0.046
% retinol in vitamin A intake		0.32	0.018
% RDA of vitamin B6	0.32		0.021
% RDA of Ca	0.40^∗∗^		0.023
Significant correlations of DSAS with food consumption/nutrient intake (*r* values)			
Whole-grain bread	0.28	—	0.036
	—	0.44	0.017
Legumes	0.24	—	0.019
	—	0.24	0.039
Tea	0.31		0.018
Apple		0.31	0.028
EPA		0.25	0.032
% energy from ALA		0.27	0.026
% energy from MUFA		0.26	0.044
% RDA of vitamin C	−0.25	—	0.041
	—	0.25^∗^	0.031
% RDA of vitamin A		0.44	0.0026
% retinol in vitamin A intake		0.55	0.0012
% RDA of Fe		0.30	0.023
% RDA of Cu		0.21	0.043
Dietary fiber		0.32	0.043

^∗^Only nonsmokers. ^∗∗^Only smokers.
